# Amoebicidal Activity of Poly-Epsilon-Lysine Functionalized Hydrogels

**DOI:** 10.1167/iovs.63.1.11

**Published:** 2022-01-07

**Authors:** Stephnie M. Kennedy, Pallavi Deshpande, Andrew G. Gallagher, Malcolm J. Horsburgh, Heather E. Allison, Stephen B. Kaye, Donald A. Wellings, Rachel L. Williams

**Affiliations:** 1 Department of Eye and Vision Science, Institute of Life Course and Medical Sciences, University of Liverpool, Liverpool, United Kingdom; 2 SpheriTech Ltd, The Heath Business and Technical Park, Runcorn, Cheshire, United Kingdom; 3 Department of Infection Biology and Microbiomes, Institute of Infection, Veterinary and Ecological Sciences, University of Liverpool, Liverpool, United Kingdom; 4 Department of Clinical Infection, Microbiology and Immunology, Institute of Infection, Veterinary and Ecological Sciences, University of Liverpool, Liverpool, United Kingdom

**Keywords:** amoebicidal, hydrogels, antitrophozoite, anticyst

## Abstract

**Purpose:**

To determine the amoebicidal activity of functionalized poly-epsilon-lysine hydrogels (pɛK^+^) against *Acanthamoeba castellanii*.

**Methods:**

*A. castellanii* trophozoites and cysts were grown in the presence of pɛK solution (0–2.17 mM), pɛK or pɛK^+^ hydrogels, or commercial hydrogel contact lens (CL) for 24 hours or 7 days in PBS or Peptone-Yeast-Glucose (PYG) media (nutrient-deplete or nutrient-replete cultures, respectively). Toxicity was determined using propidium iodide and imaged using fluorescence microscopy. Ex vivo porcine corneas were inoculated with *A. castellanii* trophozoites ± pɛK, pɛK^+^ hydrogels or commercial hydrogel CL for 7 days. Corneal infection was assessed by periodic acid–Schiff staining and histologic analysis. Regrowth of *A. castellanii* from hydrogel lenses and corneal discs at 7 days was assessed using microscopy and enumeration.

**Results:**

The toxicity of pɛK^+^ hydrogels resulted in the death of 98.52% or 83.31% of the trophozoites at 24 hours or 7 days, respectively. The toxicity of pɛK^+^ hydrogels resulted in the death of 70.59% or 82.32% of the cysts in PBS at 24 hours or 7 days, respectively. Cysts exposed to pɛK^+^ hydrogels in PYG medium resulted in 75.37% and 87.14% death at 24 hours and 7 days. Ex vivo corneas infected with trophozoites and incubated with pɛK^+^ hydrogels showed the absence of *A*. *castellanii* in the stroma*,* with no regrowth from corneas or pɛK^+^ hydrogel, compared with infected-only corneas and those incubated in presence of commercial hydrogel CL.

**Conclusions:**

pɛK^+^ hydrogels demonstrated pronounced amoebicidal and cysticidal activity against *A. castellanii*. pɛK^+^ hydrogels have the potential for use as CLs that could minimize the risk of CL-associated *Acanthamoeba* keratitis.

A*canthamoeba* keratitis (AK) is a rare infection but may result in severe visual impairment or blindness.^1^^,^^2^ *Acanthamoeba* are opportunistic protozoan, amoebic parasites that live naturally in soil, air, and aquatic habitats in one of two forms: trophozoites (mobile active form) or cysts (highly resilient, double-walled, dormant cysts).^3^ Trophozoites and cysts are both able to infect the cornea, skin, and central nervous system.^4^ The conversion of trophozoites into cysts results from adverse environmental conditions.^5^^,^^6^

The corneal epithelium normally provides a barrier to invading pathogens, but *Acanthamoeba* invade the cornea through a breech in the corneal epithelium. Contact lens (CL) wear is a significant risk factor for the development of AK, and up to 80% of cases of AK are associated with the use of CLs or lens solutions contaminated with waterborne *Acanthamoeba.* In particular, there has been an increase in AK associated with the increased use of soft CLs.^7^^–^^9^ In addition, a lack of compounds effective against *Acanthamoeba* cysts in CL solutions and tap water quality are also contributing risk factors of AK in CL wearers.^10^^–^^13^

To initiate infection, trophozoites adhere to the corneal epithelium via mannose-decorated glycoproteins and invade the cornea as enzymes such as matrix metalloproteases, serine, and cysteine proteases degrade the corneal stroma.^14^ Within the stroma, trophozoites may encyst, which may lead to a persistent, relapsing keratitis.^15^ Successful treatment of AK requires early diagnosis,^16^ but the diagnosis is challenging and may be mistaken for a bacterial, viral, or fungal keratitis. Treatment of established AK is difficult and currently reliant on intensive treatment with chlorohexidine, polyhexamethylene biguanide, brolene, or hexamide, with variable and often poor outcomes.^17^

Poly-epsilon-lysine (pεK) is a cationic peptide with intrinsic antimicrobial properties and that reportedly disrupts the cell membrane and cell wall of microbes showing broad-spectrum antimicrobial activity against Gram-positive and Gram-negative bacteria, yeasts, and fungi.^18^^–^^21^ Our previous studies have demonstrated pɛK hydrogels are effective against laboratory strains of *Escherichia* *coli*, *Staphylococcus* *aureus*, and *Pseudomonas* *aeruginosa* while having no toxicity toward corneal epithelial cells.^22^^–^^24^ Further functionalization of the free amine groups of the hydrogel with covalently bound pɛK (pɛK^+^) increased pɛK levels with a 10-fold increase in amine functionality due to additional pɛK molecules when compared with nonfunctionalized hydrogels.^22^ Further studies associated this increase in pɛK with increased antimicrobial activity against *P. aeruginosa*, reducing the number of viable bacteria in in vitro and ex vivo corneal infection models.

The aim of this study was to evaluate whether pɛK offers amoebicidal activity against *Acanthamoeba* in both trophozoite and cyst forms. The effects of pɛK solution, pɛK hydrogel, and pɛK^+^ hydrogel treatment upon both trophozoite and cyst forms of *Acanthamoeba* were investigated at 24 hours and 7 days in vitro, and significant toxicity was demonstrated with both forms. Using an ex vivo porcine cornea model of AK, we showed that no *A. castellanii* were detected in the stroma after application of pɛK^+^ or pɛK hydrogels.

## Methods

### pɛK Solution Preparation

pɛK (Bainafo; Zhengzhou Bainafo Bioengineering Co., Ltd., Zhengzhou 450006, Henan Province, China) (20 mM) was prepared in sterile PBS (Oxoid, Hampshire, UK), filter sterilized using a 0.2-µm filter, and serially diluted in twofold increments in PBS to desired concentrations.

### pɛK Hydrogel Synthesis and Functionalization

pɛK hydrogels were synthesized and functionalized as previously described.^22^^,^^24^ Briefly, pεK^+^ hydrogels were synthesized from pεK crosslinked 60 mol % with octanedioic acid to a polymer density of 0.071 g mL^–1^ using carbodiimide chemistry. Pendant pεK was covalently bound to free amine groups using 1-ethyl-3-(3-dimethylaminopropyl) carbodiimide and N-hydroxysuccinimide coupling.

### Cultivation of *Acanthamoeba*

*A. castellanii* (Douglas) Page (ATCC 30234) (ATCC, Manassas, VA, USA), originally isolated from a patient with corneal keratitis, was used in the study. Trophozoite cultures were maintained in axenic culture in ATCC Medium 712 Peptone-Yeast-Glucose (PYG) medium with additives in T-75 CELLSTAR tissue culture flasks (Greiner Bio-One, Stonehouse, UK) at 25°C, according to ATCC instructions. Trophozoites in exponential growth (72–96 hours) were centrifuged at 1000 × *g* for 10 minutes and resuspended in PYG media. Trophozoites were counted in a disposable Millicell, Neubauer hemocytometer (Millipore, Merck, UK) and adjusted to a final concentration of 2 × 10^4^ amoeba/mL in PYG media.

Cysts were prepared following centrifugation of trophozoites and pellets washed in PBS by gentle pipetting and recentrifuged to remove traces of PYG media. Trophozoites were resuspended in PBS to a final concentration of 1 × 10^6^ cysts/mL, and 1 mL of trophozoite suspension was added onto nonnutrient agar plates (3% w/v agar; Sigma-Aldrich, Dorset, UK) at 28°C for 7 to 10 days until 100% encystment was observed by microscopic analysis. Cysts were harvested using a cell scraper and resuspended in PBS containing sodium dodecyl sulfate 0.5% (w/v) (Sigma-Aldrich) to solubilize any remining trophozoites, followed by centrifugation at 1000 × g for 10 minutes and resuspended at 2 × 10^4^ cysts/mL in PBS.

### pɛK Solution Toxicity Assay Against Trophozoites and Cysts

Trophozoites and cysts were used at 2 × 10^3^ amoebae per well (100 µL) in a 96-well plate. Then, 100 µL of pɛK solutions at 2× concentration was added to each well to a final working volume of 200 µL, with a final pɛK concentration range of 0 to 4.34 mM. Chlorohexidine (CHX) (Sigma-Aldrich) at 0.02% was used as a positive control for toxicity of trophozoites and cysts. Toxicity of pɛK against *A. castellanii* was assessed using propidium iodide (PI) (Thermo Fisher Scientific, Loughborough, UK) added to wells at a final concentration of 1 µg/mL. Dead trophozoites/cysts imaged using a fluorescent microscope and the number of dead red-labeled *A. castellanii* were compared with the number of nonstained trophozoites and cysts per field of view expressed as percentage dead (%) compared with total live and dead.

### pεK Cytotoxicity Against Human Corneal Epithelial Cells

Human corneal epithelial (hCE-T) cells (donated by Kaoru Araki-Sasaki, Kansai Medical University, Moriguchi, Japan)^25^ were cultured at 37°C and 5% CO_2_. hCE-T cells were cultured in Dulbecco's modified Eagle's medium/Ham's F12 media (DMEM/F12) (Thermo Fisher Scientific) containing 5% (v/v) fetal calf serum (Biosera; Labtech, Heathfield, UK) with 1% (v/v) fungizone and penicillin/streptomycin (Sigma-Aldrich) supplementation. hCE-T cells were seeded in 96-well plates at 1 × 10^4^ hCE-T cells and incubated for 3 days until 80% confluence was achieved. Media were replaced with fresh media containing pɛK (0–17.36 mM) and incubated at 37°C and 5% CO_2_ for 24 hours and 7 days. At each time point, viability/toxicity assays were performed using the Live/Dead viability/cytotoxicity kit for mammalian cells (Thermo Fisher Scientific) following the manufacturer's instructions. hCE-T cells were incubated with Calcein-AM and ethidium homodimer 1 for 30 minutes and fluorescent live (green)/dead (red) cells imaged using an imaged Zeiss Apotome (Germany) live-cell microscope.

### pɛK Hydrogel Toxicity Assay Against Trophozoites and Cysts

Under sterile conditions, 10-mm discs of pɛK and pɛK^+^ hydrogels or commercial hydrophilic cast-molded CL (Hydrogel CL; Filcon II 2, 77% water content; Ultravision, Leighton Buzzard, UK) were added to 48-well plates using sterile forceps. Trophozoites or cysts were used at 1 × 10^4^ amoebae per well in 500 µL of PBS (nutrient deplete) or PYG media (nutrient replete) and incubated at 28°C for either 24 hours or 7 days. Toxicity of pɛK hydrogels was measured as described above following the addition of 1 µg/mL propidium iodide to label dead trophozoites and cysts.

### Ex Vivo Porcine Cornea Keratitis Model

Porcine eyes were obtained from a local abattoir within 6 hours of slaughter, and corneas were processed with agarose supports to establish an air–liquid interface ex vivo organ culture model as previously described by Kennedy et al.^23^^,^^24^ and Supplementary 1. Trophozoites were suspended in 10 µL PYG media containing 1 × 10^5^ amoebae and pipetted onto an air-dried region of the central cornea and allowed to absorb for 30 minutes at room temperature (assessed by visual analysis). Once the droplet was no longer visible, 10-mm discs of commercial CL, pɛK, or pɛK^+^ hydrogels were applied to corneas. Control infected and noninfected corneas were run in parallel. DMEM (+10% FBS) was added up to the scleral boundary of the cornea (∼3 mL) and incubated for 7 days at 37°C, 5% CO_2_.

Following infection, pɛK^+^ and pɛK hydrogel lenses and commercial hydrogel CLs were removed from corneas, resuspended in PYG media, and vortexed for 30 seconds to remove *A. castellanii* from lenses, and the solution was transferred to a 6-well plate to monitor regrowth. Quantification of *A. castellanii* infection of corneas was achieved by trephining (Blink Medical Ltd, Solihull, UK) 10-mm discs from the central cornea. To isolate trophozoites, corneal discs were cut into quadrants and further quartered, resuspended in 3 mL PBS, and briefly vortexed, followed by homogenization using a Qiagen TissueRuptor (Qiagen, Manchester, UK) for 15 seconds. The homogenate was centrifuged at 1000 × *g* for 10 minutes, resuspended in PYG media (+ glucose 1.5% (w/v)), transferred to a well of a 6-well plate, and cultured for 7 days to monitor regrowth. *A. castellanii* from each experimental condition were imaged at 7 days using a Nikon (Nikon Europe BV, Netherlands) Ti-E microscope and quantified following resuspension of *A. castellanii* in PYG media using a hemocytometer.

### Histology

A separate set of corneas from each experimental condition was fixed in 10% (v/v) neutral buffered formalin (Sigma-Aldrich) for 18 hours. Corneas were processed using a Leica ASP300 (Nussloch, Germany) tissue processor. Paraffin-embedded tissue was sectioned at a thickness of 5 µm and stained with periodic acid–Schiff (PAS) (Abcam, Cambridge, UK) following the manufacturer's protocol for positive detection of chitin in the cell wall of *A. castellanii.* Tissue sections were imaged using a Nikon CI upright microscope using a 20× and 40× objective.

### Statistical Analysis

Experiments were performed in triplicate (*n* = 3), with three wells per experiment and five fields of view in each well. One-way ANOVA was performed with a post hoc Tukey's analysis, and *P* < 0.05 was considered significant. Statistical analysis was performed using Prism software version 8.02.263 (GraphPad Software, La Jolla, CA, USA). Error bars are shown as standard deviations of biological replicates.

## Results

### Amoebicidal, Both Trophozoicidal and Cysticidal, Activity of pɛK Solution Against *A. castellanii*

Trophozoicidal and cysticidal effects of pɛK solution (0–4.34 mM) on *A. castellanii* were assessed following incubation for 24 hours and 7 days. Toxicity was assessed by the number of dead, red PI-stained *A. castellanii* compared to nonstained live *A. castellanii* and expressed as percentage death at each dose.

**Figure 1.**
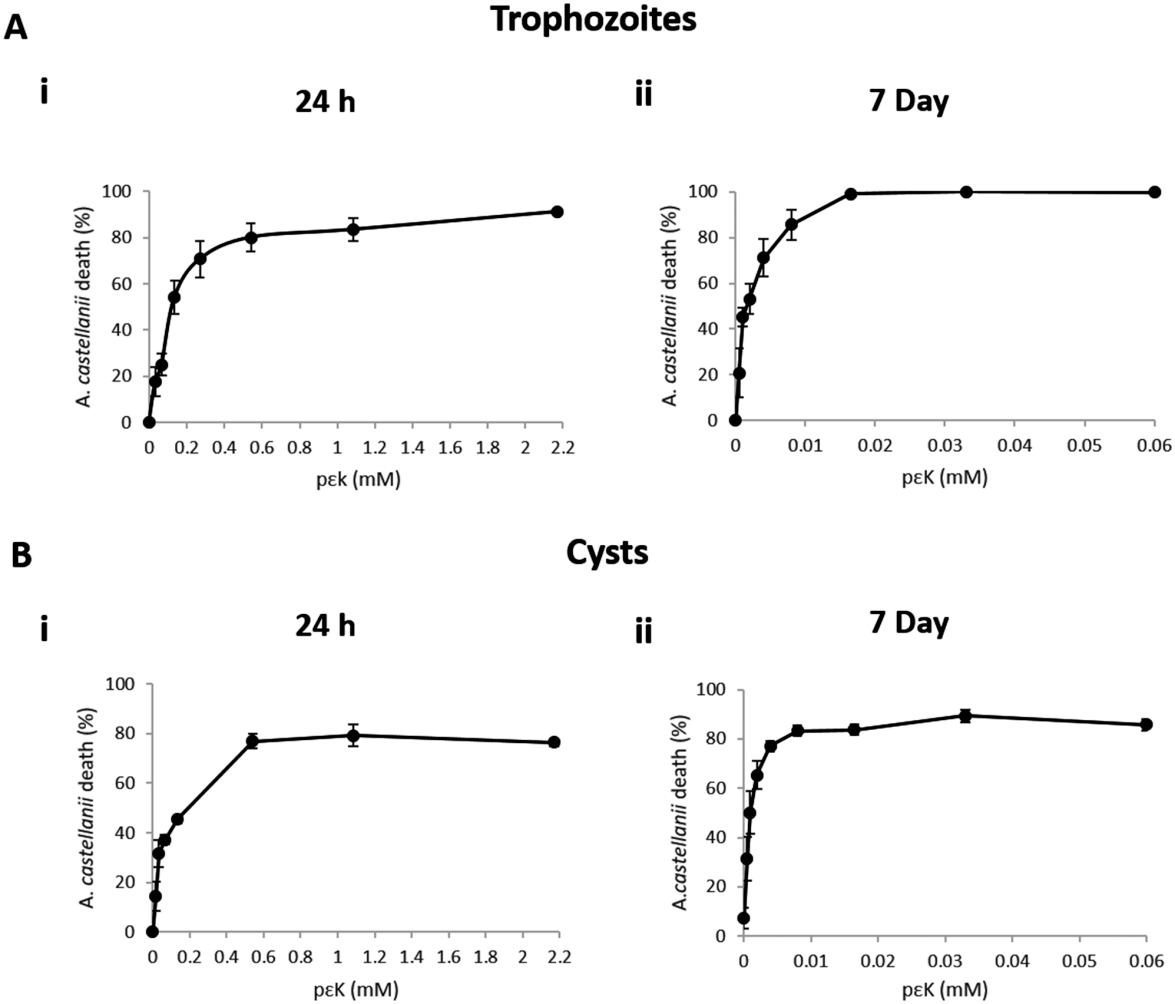
Dose response of pɛK solutions against *A. castellanii* trophozoites and cysts at 24 h and 7 days. (**A**) Quantification of the percentage dead trophozoites compared to total trophozoites identified by PI staining at (i) 24 h and (ii) 7 days. (**B**) Quantification of the percentage dead cysts identified by PI staining at (i) 24 h and (ii) 7 days.

After 24-hour pɛK treatment of trophozoites, 80% (SD ± 6.14%) toxicity was observed at 0.54 mM pɛK (*P* < 0.0001) compared to untreated trophozoites, and no further increase in toxicity above 80% was achieved at any higher concentrations (Fig. 1Ai). After 24 hours, trophozoites remained attached to the tissue culture plastic surface (TCPS) surface showing characteristic morphology, while treatment with CHX showed 100% toxicity. An increased dose of pɛK increased the number of dead *A. castellanii*, while live *A. castellanii* detached from the surface and did not show characteristic trophozoite morphology. At 7 days posttreatment, amoebicidal effects of pɛK upon trophozoites were observed at lower pɛK doses (Fig. 1Aii). After 7-day treatment with lower concentrations of pɛK (0.016 mM), the number of dead trophozoites increased to 99% (SD ± 1.04%) (*P* < 0.0001).

Effects of pɛK solution upon cysts after 24-hour treatment showed 76% (SD ± 1.50%) cyst death at 0.54 mM and above (*P* < 0.0001) (Fig. 1Bi). At 7 days (Fig. 1Bii), treatment with pɛK solution led to increased number of dead cysts at lower pɛK concentrations. Toxicity against cysts reached 77% (SD ± 2.14%) at 0.004 mM (*P* < 0.0001), and cyst death did not significantly increase (*P* < 0.05) further with increased pɛK concentrations. At 24 hours and 7 days, only live cysts were visible in control images, while in comparison, CHX showed red PI-stained cysts in the field of view.

### Toxicity of pɛK Solution Against hCE-T Cells

Maximum toxicity of pɛK treatment toward trophozoites and cysts was observed at 0.54 mM at 24 hours and at 0.016 mM and 0.004 mM at 7 days for trophozoites and cysts, respectively. Toxicity of pɛK solution at different concentrations was determined upon confluent monolayers of hCE-T cells.

**Figure 2.**
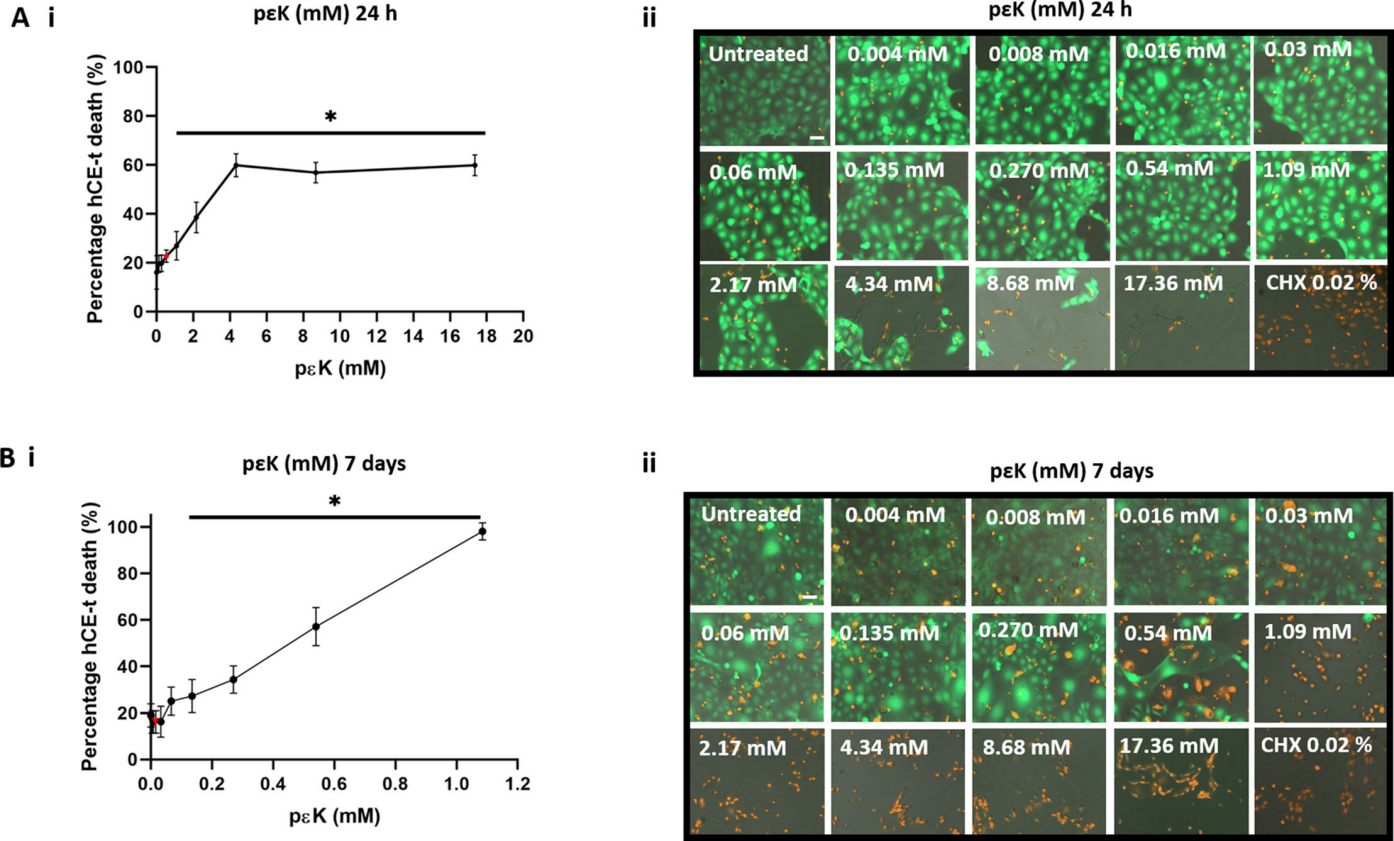
Dose response of pɛK solutions against human corneal epithelial cell line hCE-T at 24 hours and 7 days. pɛk solutions (0–17.36 mM) added to hCE-T cells and dead cells were labelled with PI (*red*) and live cells labeled with calcein AM (*green*). (**A**) (i) Quantification of the percentage dead hCE-T cells in the total population at 24 hours. The *red* data point indicates the toxic dose of pɛK toward trophozoites and cysts. (ii) Corresponding fluorescent images of hCE-T cells; dead cells were labeled with PI (*red*) and live cells labeled with calcein AM (*green*). (**B**) (i) Quantification of the percentage dead hCE-T cells in the total population at 7 days. The *red* data point indicates the toxic dose of pɛK toward trophozoites and cysts. (ii) Corresponding fluorescent images of hCE-T cells; dead cells were labeled with PI (*red*) and live cells labeled with calcein AM (*green*). *Scale bars*: 50 µm.

After 24-hour treatment with pɛK solution, confluent monolayers of live hCE-T cells (stained green with calcein-AM dye) were observed after application up to 0.54 mM pɛK**,** with comparable dead hCE-T cells (stained red with PI) to untreated controls (*P* > 0.05) (Figs. 2Ai, Aii). At 1.09 mM pɛK and above, live cells were reduced, with increased dead cells per image compared to untreated controls (*P* < 0.05). At 7 days, treatment with pɛK 0 to 0.067 mM was not toxic to hCE-T cells (*P* > 0.05) and, at 0.135 mM and above, proved toxic to hCE-T cells with a reduction in live hCE-T cells (*P* < 0.05) (Figs. 2Bi, Bii).

### Trophozoicidal Activity of pɛK Hydrogels Against *A.* *castellanii*

**Figure 3.**
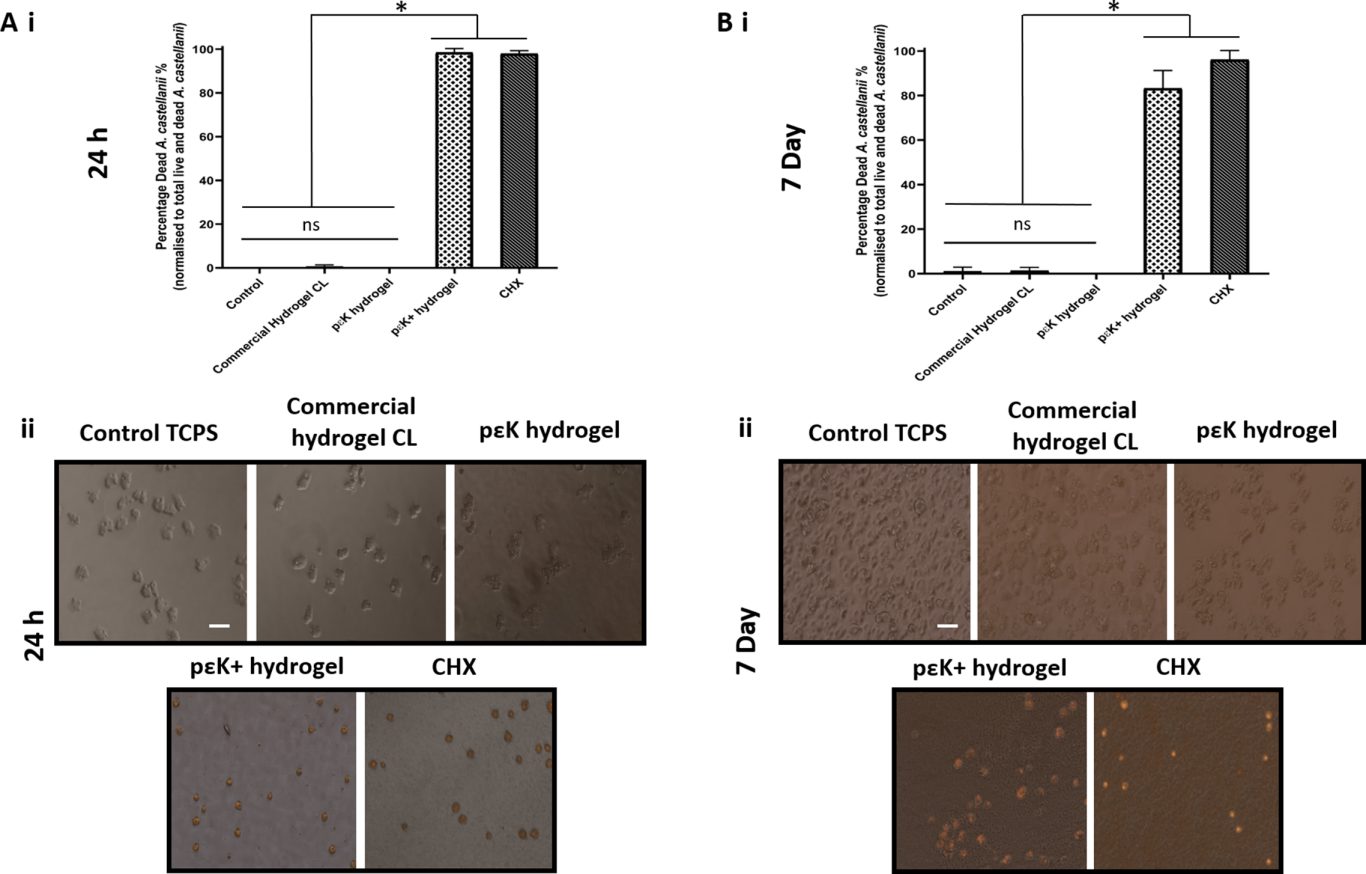
Toxicity of pɛK^+^ hydrogels against *A. castellanii* trophozoites at 24 hours and 7 days. (**A**) (i) graph to show the percentage of dead trophozoites and (ii) fluorescent images of trophozoites when cultured on TCPS, commercial hydrogel CL, and pɛK and pɛK^+^ hydrogels for 24 hours. (**B**) (i) Graph shows the percentage of dead trophozoites to total live and dead trophozoites and (ii) fluorescent images of trophozoites when cultured on TCPS, commercial hydrogel CL, and pɛK and pɛK^+^ hydrogels for 7 days. CHX was run as a positive control for trophozoite death. *Scale bars*: 50 µm.

Toxicity of pɛK^+^ hydrogels toward trophozoites at 24 hours and 7 days was assessed. At 24 hours, trophozoites grown on TCPS, commercial hydrogel CL, and nonfunctionalized pɛK hydrogels adhered to the surface, with few cysts forming and minimal trophozoite death, identified by PI staining in <1% of the population (Figs. 3Ai, Aii) with no significant difference compared to untreated controls. Trophozoites cultured on pɛK^+^ hydrogels, however, showed 98.52% (SD ± 1.82%) (*P* < 0.0001) death, similar to CHX (97.93% [SD ± 1.38%]; *P* < 0.0001) (Fig. 3Aii), compared to untreated controls, commercial CLs, and pɛK hydrogels. There was no significant difference in death between pɛK^+^ hydrogels and CHX (*P* = 0.094).

At 7 days, growth on pɛK^+^ hydrogels showed 83.31% (SD ± 7.96%) toxicity against trophozoites compared to untreated controls (*P* < 0.0001). The total number of live and dead trophozoites decreased by 7 days compared to 24 hours, with dead trophozoites being degraded within the media, accounting for the lower percentage reduction. In comparison, trophozoites cultured on nonfunctionalized pɛK hydrogels, commercial hydrogel CL, and TCPS all showed growth of trophozoites with negligible toxicity <1% in the total population (Figs. 3Bi, Bii), with no significant difference (*P* < 0.05) compared to untreated controls. Treatment with CHX showed 96.01% (SD ± 4.12%) dead trophozoites and was slightly more effective compared to pɛK^+^ hydrogels (*P* = 0.024).

### Cysticidal Activity of pɛK Hydrogels Against *A.* *castellanii*

**Figure 4.**
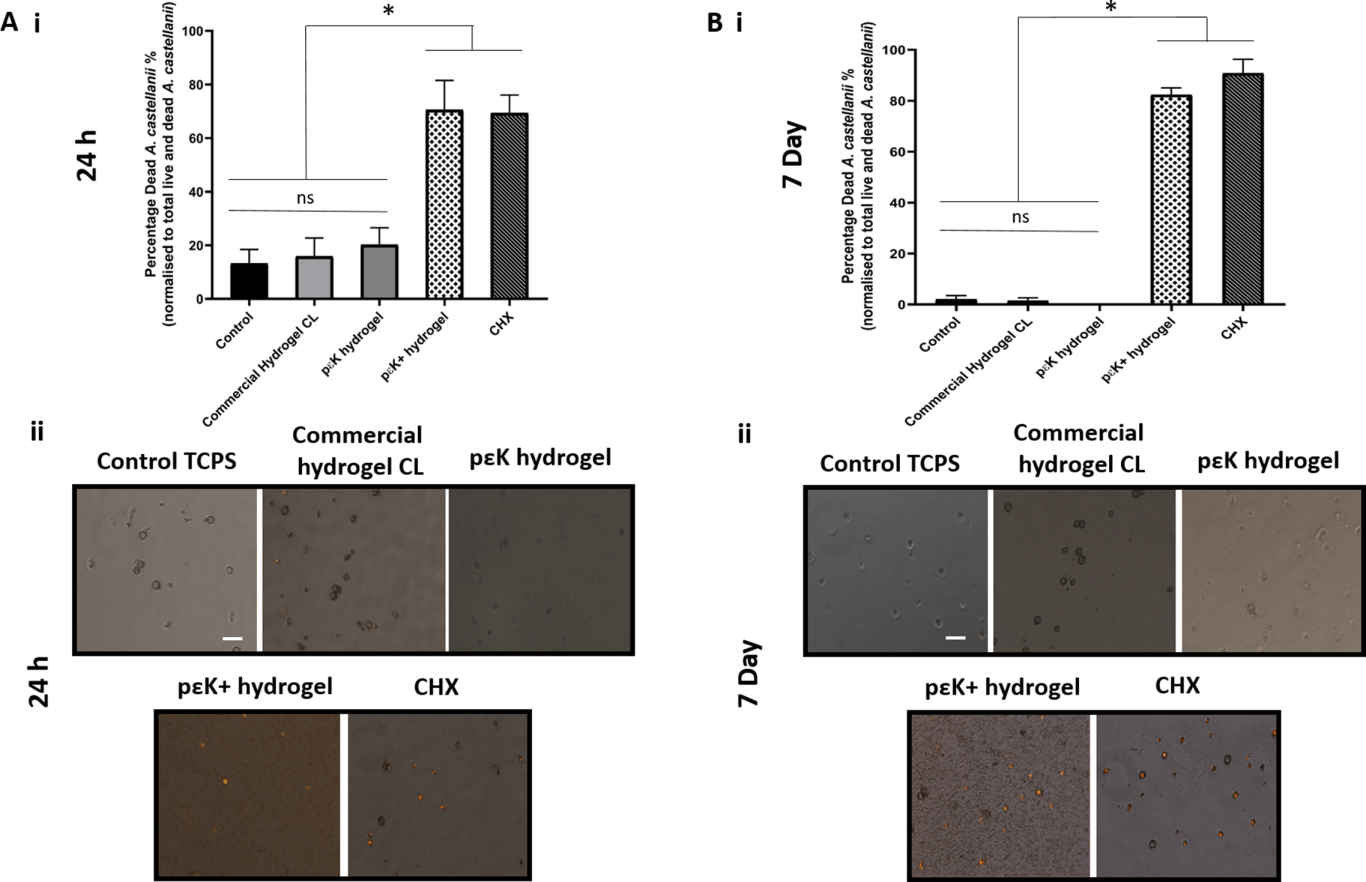
Toxicity of pɛK^+^ hydrogels against *A. castellanii* cysts cultured in PBS buffer (nutrient deplete) at 24 hours and 7 days. (**A**) (i) Graph shows the percentage of dead cysts and (ii) fluorescent images of cysts when cultured on TCPS, commercial hydrogel CL, and pɛK and pɛK^+^ hydrogels for 24 hours. (**B**) (i) Graph shows the percentage of dead cysts to total live and dead cysts and (ii) fluorescent images of cysts when cultured on TCPS, commercial hydrogel CL, and pɛK and pɛK^+^ hydrogels for 7 days. CHX was run as a positive control for cyst death. *Scale bars*: 50 µm.

Cysts were cultured on TCPS, pɛK and pɛK^+^ hydrogels, and commercial hydrogel CLs in both nutrient-rich PYG medium or nonnutrient PBS to mimic dormant or proliferating conditions for 24 hours or 7 days. Cysts cultured in PBS remained in cyst form on TCPS, pɛK or pɛK^+^ hydrogels, or commercial hydrogel CLs. At 24 hours under control untreated conditions, the percentage of dead cysts in the overall population was 13.18% (SD ± 5.29%). There was an increase in dead cysts when cultured on pɛK^+^ hydrogels of 70.59% (SD ± 10.93%) and CHX of 69.37% (SD ± 6.68%), both significantly different from untreated controls, pɛK hydrogels, or commercial CL (*P* < 0.0001) (Fig. 4A). After 7 days, dead cysts increased to 82.32% (SD ± 2.74%) on the pɛK^+^ hydrogel, and CHX was slightly more effective with 90.83% (SD ± 5.45%) (Fig. 4B) (*P* = 0.03), with both resulting in significantly higher cysticidal activity compared to untreated controls, pɛK hydrogels, and commercial CLs (*P* < 0.0001).

**Figure 5.**
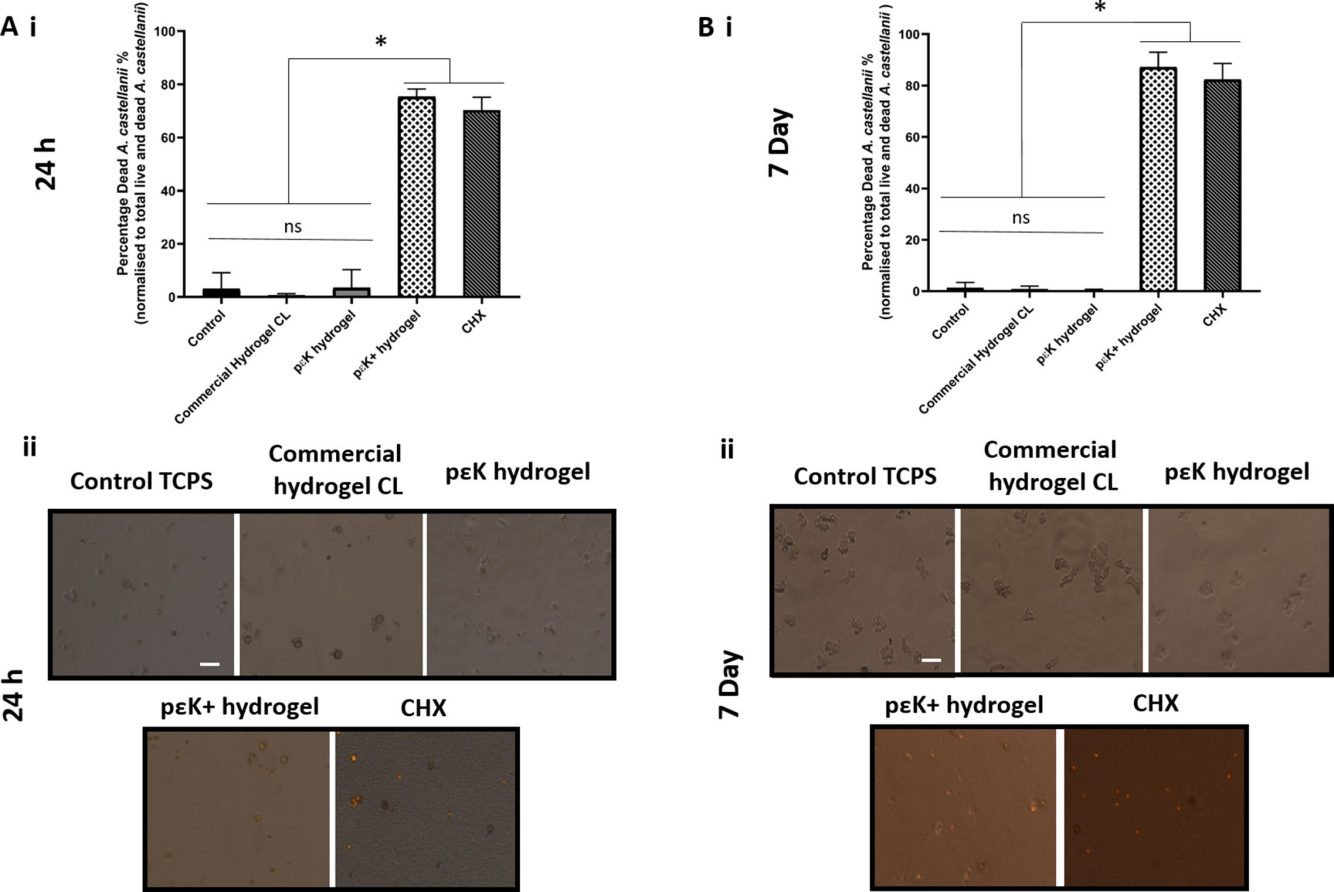
Toxicity of pɛK^+^ hydrogels against *A*. *castellanii* cysts cultured in PYG buffer (nutrient replete) at 24 hours and 7 days. (**A**) (i) Graph shows the percentage of dead cysts to total live and dead cysts and (ii) fluorescent images of cysts when cultured on TCPS, commercial hydrogel CL, and pɛK and pɛK^+^ hydrogels for 24 hours. CHX was run as a positive control for cyst death. (**B**) (i) Graph shows the percentage of dead cysts and (ii) fluorescent images of cysts when cultured on TCPS, commercial hydrogel CL, and pɛK and pɛK^+^ hydrogels for 7 days. CHX was run as a positive control for cyst death. *Scale bars*: 50 µm.

Cysts cultured in PYG medium on TCPS, commercial hydrogel CL, and pɛK hydrogels differentiated into trophozoites at 24 hours (Fig. 5A). In contrast, cysts cultured in PYG medium on pɛK^+^ hydrogels did not differentiate into trophozoites and showed 75.37% (SD ± 2.84%) reduction in viable cysts with no significant difference compared to CHX (70.27% [SD ± 4.84%]) (*P* = 0.68). Both pɛK^+^ hydrogel and CHX were significantly different from untreated controls, pɛK^+^ hydrogel, and commercial hydrogel CL (*P* < 0.0001). By 7 days, dead cysts on pɛK^+^ hydrogel had increased to 87.14% (SD ± 5.79%), comparable to CHX with 82.36% (SD ± 6.24%) (*P* = 0.69), and were both significantly different from untreated controls, pɛK hydrogel, and commercial hydrogel CL (*P* < 0.0001).

### Toxicity of pɛK Hydrogels Against *A.* *castellanii* in Ex Vivo Porcine Corneas

**Figure 6.**
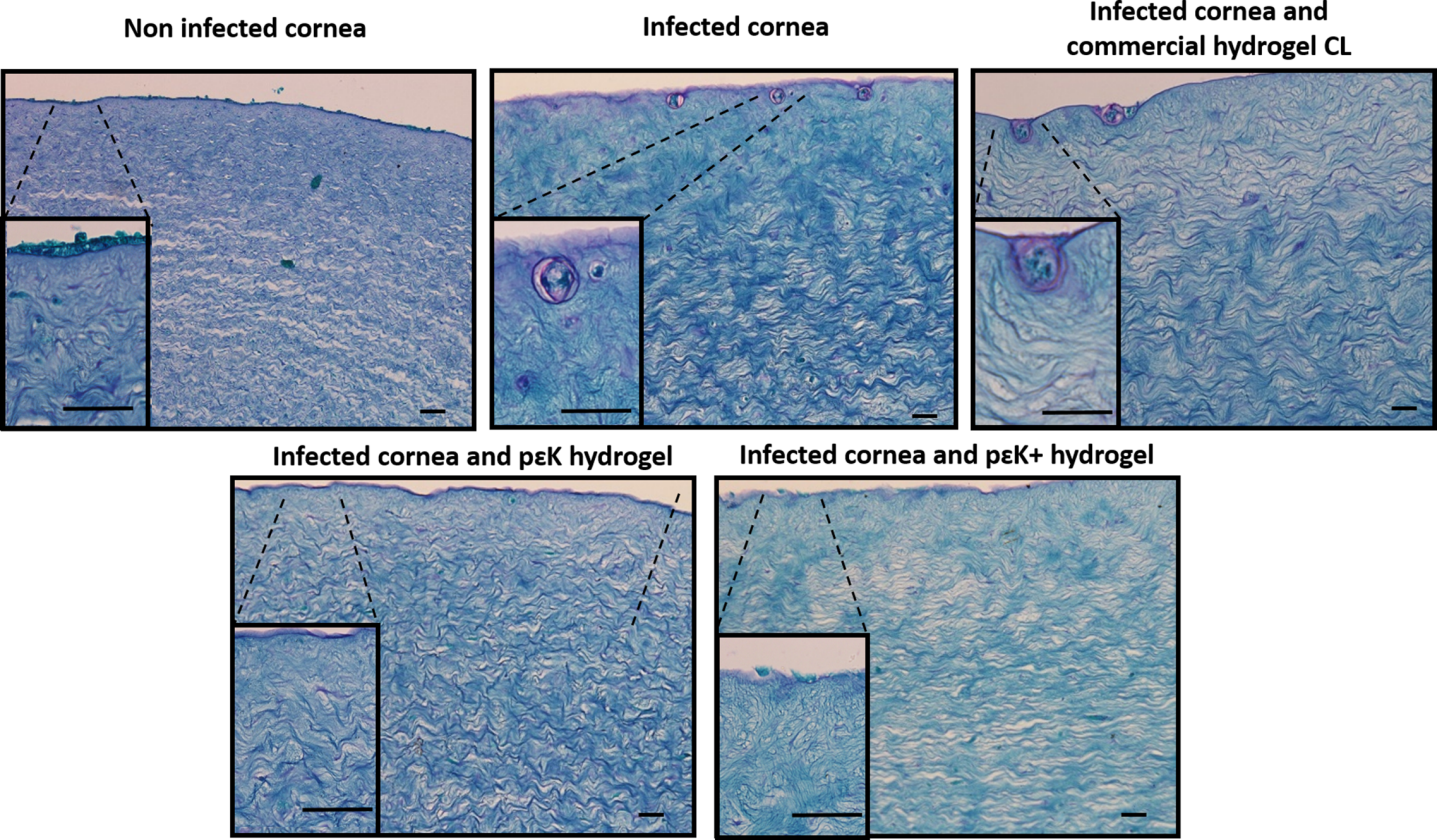
Ex vivo corneal infection of *A. castellanii* trophozoites in the presence of pɛK^+^ hydrogels. Histologic sections of PAS staining of porcine corneas infected with *A*. *castellanii* trophozoites for 7 days. *Top row left* shows no infection in cornea, *middle top* shows infected cornea (insert highlighting *A*. *castellanii* cyst), and *top right* shows A. *castellanii* grown in the presence of a commercial hydrogel CL. *Bottom row* shows corneas infected with *A*. *castellanii* in the presence of pɛK hydrogel and pɛK^+^ hydrogels. *Scale bars*: 50 µm.

Porcine corneas were infected with trophozoites for 7 days in the presence and absence of commercial hydrogel CL, pɛK hydrogel, or pɛK^+^ hydrogel (Fig. 6). Histologic analysis of fixed corneas using PAS to label *A. castellanii* showed there were no *A. castellanii* within the corneal stroma in uninfected corneas. Infected corneas and infected in the presence of commercial hydrogel CL both showed cysts below the corneal surface. Infected corneas incubated with pɛK or pɛK^+^ hydrogels showed an absence of *A. castellanii* within tissue sections.

**Figure 7.**
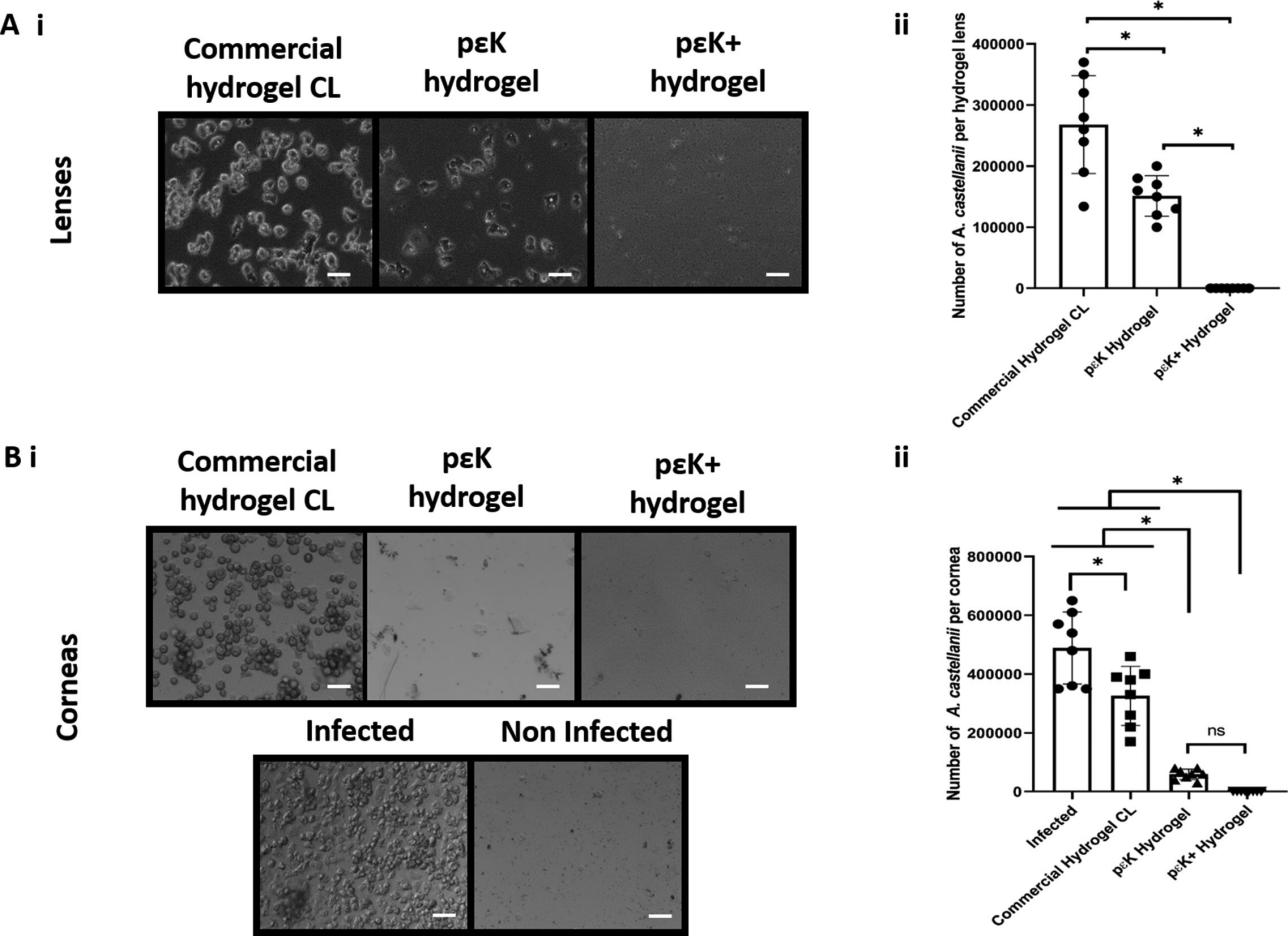
Growth of *A. castellanii* from pɛK^+^ hydrogels and corneas from ex vivo infection model. (**A**) Lenses were removed from corneas and cultured in PYG media for 7 days. (i) phase contrast images of *A*. *castellanii* regrowth from lenses and (ii) quantification at 7 days. (**B**) Regrowth of *A. castellanii* from cornea (i) phase contrast images and (ii) quantification at 7 days. *Scale bars*: 50 µm.

Numeration of *A. castellanii* present on lenses after incubation with corneas showed trophozoite regrowth at 7 days from commercial hydrogel CL (2.68 × 10^5^ amoebae) and pɛK hydrogel (1.51 × 10^5^ amoebae) (*P* = 0.0004) (Figs. 7Ai, Aii). No detectable viable *A. castellanii* retrieved were from the pɛK^+^ hydrogel, which was significantly different from other hydrogel lenses (*P* < 0.0001).

After 7 days, regrowth of *A. castellanii* from homogenized corneal buttons from infected corneas and corneas from under commercial hydrogel CL showed 4.89 × 10^5^ and 3.12 × 10^5^ amoebae per cornea, respectively (*P* = 0.0018) (Figs. 7Bi, Bii). *A. castellanii* regrowth from corneas under pɛK hydrogel was 5.88 × 10^4^ amoebae per cornea, compared to infected and commercial hydrogel CL (*P* < 0.0001). No detectable *A. castellanii* were retrieved from corneas incubated with pɛK^+^ hydrogel, which was significantly different compared to infected and commercial hydrogel CL (*P* < 0.0001) corneas but not from pɛK hydrogel corneas (*P* = 0.47).

## Discussion

This study demonstrated the antimicrobial peptide pɛK is effective at reducing viable *A. castellanii* trophozoites and cysts, both as a solution and when covalently bound to pɛK hydrogels using in vitro assays. pɛK was nontoxic to hCE-T cells at doses up to 0.54 mM at 24 hours and up to 0.067 mM at 7 days, incorporating doses that were toxic to *A. castellanii*. pɛK^+^ hydrogels demonstrated toxicity toward both cysts and trophozoites compared to nonfunctionalized pɛK hydrogels and commercial hydrogel CLs. In our ex vivo porcine corneal infection model, pɛK and pɛK^+^ hydrogels prevented infection of *A. castellanii* within the cornea stroma, and reduced numbers of *A. castellanii* adhered to hydrogels.

Current preventative treatments against AK are targeted at maintaining good hygiene regimes^15^ and use of disinfectants within CL solutions. Therapeutic treatments for established AK involve the use of active reagents such as polyhexamethylene biguanide (0.02%) (or CHX 0.02%) and propamidine (0.1%) and hexamidine (0.1%), often in combination.^15^^,^^26^^,^^27^ Increased resistance to many of these reagents has been reported in different strains of *Acanthamoeba*.^28^^,^^29^ We have investigated only one particular strain of *Acanthamoeba*, but we have examined the effectiveness of pɛK^+^ hydrogels at killing both trophozoites and cysts, the latter of which are particularly difficult to kill. MeniCare Pure CL solution (Menicon Ltd., Nagoya, Japan) contains pεK as an active ingredient, which is effective against *Acanthamoeba.* It is used as a disinfectant instead of polyhexamethylene biguanide, but this CL solution is only suitable for use with rigid gas-permeable CLs.

The effects of pɛK solution against trophozoites and cysts were investigated in both nutrient-rich and nutrient-deplete environments. Toxic effects of pɛK against *A. castellanii* occurred in a dose- and time-dependent manner against both cysts and trophozoites in nutrient-replete and nutrient-deplete environments. The effects of pεK were more effective against the trophozoites, which are easier to treat compared to the cysts.^30^^–^^32^

Current treatments for prevention or treatment of an AK are typically harsh and damaging to the ocular surface, in particular the corneal epithelium.^33^ We demonstrated that effective doses of pεK were toxic against trophozoites or cysts, respectively, but not to hCE-T cells. pεK is generally regarded as safe, is used in many applications,^18^ and offers potential use as a treatment for AK.

There are currently no commercial CLs available that offer antimicrobial activity, and CLs themselves provide a potential route of infection into the cornea. Having established pεK solution was toxic to *A. castellanii*, we demonstrated pɛK^+^ hydrogels showed toxicity at 24 hours and 7 days toward *A. castellanii*, compared to the commercial CLs tested. pɛK^+^ hydrogels offer inherent antiamoebicidal (both trophozoicidal and cysticidal) activity without the potential need for topical application*.* CLs themselves provide a potential surface for *Acanthamoeba* to proliferate, and trophozoites and cysts are reported to adhere to all types of lenses.^34^^–^^38^ Studies have shown *Acanthamoeba* possess an affinity for attachment to CLs (in particular soft CL) contributing toward development of AK.^9^^,^^39^^,^^40^ Lee et al.^7^^,^^8^ demonstrated that rigid gas-permeable CLs with a smoother, waxier, and more homogeneous surface showed decreased adhesion of *Acanthamoeba.* The water content and mobility of CLs are important factors in the adhesion of *Acathamoeba.*^41^^,^^42^ Our study showed that *A. castellanii* can adhere and proliferate on the surface of the commercial CLs, whereas the pɛK^+^ hydrogel reduced the number of viable *A. castellanii*.

In addition, the ex vivo infection model demonstrated pεK^+^ hydrogels prevented infection by *A. castellanii* into the corneal stroma with no cyst formation, as was seen with commercial CLs. There was also a reduced recovery of *A. castellanii* from the pεK^+^ hydrogels compared to commercial CLs or infected corneas. Teuchner et al.,^43^ using a porcine corneal ex vivo model, reported at 7 days there was deeper penetration of *Acanthamoeba* into stroma compared to 4 days with higher infection using PYG media compared to PBS. We used DMEM to ensure nutrients were supplied to the cornea during the experiment; it is not clear, therefore, if we would have observed higher rates of infection with PYG, supplying a nutrient-rich media for *Acanthamoeba* growth.

Antimicrobial effects of pεK are well documented, and our studies have reported antimicrobial effects of pεK^+^ hydrogels toward Gram-positive and Gram-negative bacteria as well as fungi.^22^^,^^24^^,^^44^^,^^45^ *Acanthamoeba* predate upon bacteria and fungi, which may be transported within *Acanthamoeba*, leading to a coinfection.^46^^–^^48^ pεK hydrogels offer the ability to provide effective treatment against multiple microbes, due to functionalization of the free amine group on pεK providing innate antimicrobial properties. The current proposed mechanism of action for pεK involves the positively charged pεK peptide interacting with negatively charged cell membranes, leading to cell lysis.^18^

AK is a rare but serious condition leading to visual loss, with an increase in number of cases associated with increased use of CLs.^10^^,^^49^ Infection in non-CL wearers can also occur when the integrity of the ocular surface is compromised through surgery or trauma or if patients are immunocompromised.^49^^–^^51^ CLs are important medical devices for visual and therapeutic purposes but pose a significant risk factor in the development and progression of AK.^7^^,^^13^ Diagnosis and treatment of AK are difficult, and treatments that offer toxicity toward *Acanthamoeba* are limited, with many only effective against trophozoites and not cysts.^10^ There is therefore a need for development of new therapies and approaches to prevent and treat AK and combat resistance to current *Acanthamoeba* treatments.^52^ In summary, our data show pεK^+^ hydrogels offer amoebicidal and cysticidal activity against *A. castellanii*, which could potentially lessen the risk of CL-associated AK.

## Supplementary Material

Supplement 1
