# Supplementary material for: Amoebicidal Activity of Poly-Epsilon-Lysine Functionalized Hydrogels
Source: Invest Ophthalmol Vis Sci. 2022 Jan 7;63(1):11. doi: 10.1167/iovs.63.1.11 (PMC8742527; doi:10.1167/iovs.63.1.11)
Supplement: Supplement 1 [file iovs-63-1-11_s001.pdf]

Supplementary 1

Ex vivo corneal culture

Fresh porcine eyes were obtained from 6-month old pigs within 6 h of slaughter from a local abattoir and corneas were excised as previously described.[1] Briefly, excess tissue was removed from eyes, followed by a 2 min Phosphate Buffered Saline (PBS; Oxoid) wash (containing 1% v/v penicillin/streptomycin and amphotericin B (P/S/AmpB) (Sigma, UK) Eyes containing visible lacerations identified using 2 % (w/v) Fluorescein sodium (Bausch & Lomb, Kingston-upon-Thames, UK) were excluded from the study. Corneas were washed for 2 min in sterile PBS containing 1 % (v/v) penicillin/streptomycin/amphotericin B (Sigma Aldrich, Dorset, UK), followed by a 2 min wash in 3 % (v/v) Iodinated Povidone (Ecolab Ltd, Leeds, UK) and washed thoroughly in antibiotic free PBS.

Corneas were excised from whole globes making an incision 5 mm from the limbus, followed by the removal of the iris and ciliary body tissue. The cornea was cleaned 2-3 times in fresh PBS containing 1% (v/v) P/S/AmpB to remove any unwanted cells. Corneas were placed epithelial side down into sterile bijou tube lids. UltraPure Agarose (Thermo Fisher Scientific, Loughborough, UK) (0.5 % (w/v)) dissolved in Dulbecco's Modified Eagles Medium (DMEM) (~65.5°C), cooled to ~37°C and pipetted onto the endothelial side of corneas to fill the cavity and solidified at room temperature (~25°C). Corneas and agarose supports were transferred into 6 well plates, epithelial side up, containing 3 ml DMEM (antibiotic free, containing 10% (v/v) Fetal Bovine Serum (Labtech, Heathfield, UK)) and incubated at 37°C in 5 % CO_2 for 24 h prior to infection, to ensure they were antibiotic and infection-free prior to the start of the assay.[2]

Prior to infection, corneal epithelia were debrided using sterile 6 mm filter paper discs (Grade AA Discs, Whatman®, Maidstone, UK) soaked in 70% (v/v) EtOH placed onto cornea for 5 s, followed by removal of epithelium with a surgical blade. Corneas were rinsed in DMEM and air-dried in a laminar flow cabinet for 10 min prior to inoculation with *A. castellanii* infection.

References

1 Kennedy S, Lace R, Carserides C, *et al.* Poly-epsilon-lysine based hydrogels as synthetic substrates for the expansion of corneal endothelial cells for transplantation. J Mater Sci Mater Med 2019;30:102.

2 Pinnock A, Shivshetty N, Roy S, *et a*l. Ex vivo rabbit and human corneas as models for bacterial and fungal keratitis. Graefes Arch Clin Exp Ophthalmol 2017;255:333-342.
